# Predictors of long-term stability of maxillary dental arch dimensions in patients treated with a transpalatal arch followed by fixed appliances

**DOI:** 10.1186/s40510-015-0094-9

**Published:** 2015-07-28

**Authors:** Gaetana Raucci, Maryam Elyasi, Camila Pachêco-Pereira, Vincenzo Grassia, Fabrizia d’Apuzzo, Carlos Flores-Mir, Letizia Perillo

**Affiliations:** Multidisciplinary Department of Medical-Surgical and Dental Specialties, Post-Graduate Orthodontics Program and Operative Unit, Second University of Naples, Via Luigi De Crecchio 6, 80138 Naples, Italy; School of Dentistry, University of Alberta, Edmonton, Alberta Canada

## Abstract

**Background:**

The aim of this retrospective study was to identify which dental and/or cephalometric variables were predictors of long-term maxillary dental arch stability in patients treated with a transpalatal arch (TPA) during the mixed dentition phase followed by full fixed appliances in the permanent dentition.

**Methods:**

Thirty-six patients, treated with TPA followed up by full fixed appliances, were divided into stable and relapse groups based on the long-term presence or not of relapse. Intercuspid, interpremolar and intermolar widths, arch length and perimeter, crowding, and upper incisor proclination were evaluated before treatment (*T*_0_), post-TPA treatment (*T*_1_), post-fixed appliance treatment (*T*_2_), and a minimum of 3 years after full fixed appliances’ removal (*T*_3_). A binary logistic regression was performed thereafter to evaluate the impact of the dental arch and cephalometric measurements at *T*_1_ and the changes between *T*_0_ and *T*_1_ as predictive variables for relapse at *T*_3_.

**Results:**

The proposed model explained 42.7 % of the variance in treatment stability and correctly classified 72.2 % of the sample. Of the seven predictive variables, only upper anterior crowding (*p* = 0.029) was statistically significant. For every millimeter of decreased crowding at *T*_1_ (after TPA treatment/before starting the fixed orthodontic treatment), there was an increase of 3.57 times in the odds of having stability.

**Conclusions:**

The best predictor of relapse was maxillary crowding before treatment. The odds of relapse increase by 3.6 times for every millimeter of crowding at baseline.

## Background

Non-extraction orthodontic treatments are becoming more popular nowadays. However, in patients with borderline crowding (less than 6 mm), the decision to increase the available space mainly through dental arch expansion is still questionable as its stability is uncertain [1–6].

A treatment option in class I and class II malocclusions with mild to moderate crowding and concomitant molar rotations is the use of a transpalatal arch (TPA). The TPA can relieve crowding in the upper arch primarily through molar derotation along with mild transversal dental expansion, thus inducing both increased arch width and perimeter. Additionally, during molar derotation, transeptal fibers could potentially move deciduous molars or premolars buccally, potentially offering further increases in dental arch width and perimeter [7, 8]. Nevertheless, the long-term stability of these changes remains controversial.

Only a few studies evaluated the long-term changes in upper arch after non-extraction treatment without a concomitant rapid palatal expansion process. Ciger et al*.* [9] evaluated changes in dental arches in class II division 1 malocclusion patients after non-extraction treatment with cervical headgear and full fixed orthodontic appliances. They reported that the maxillary crowding decreased during treatment by 5.5 mm but increased (relapse) after the retention stage by 3 mm. Raucci et al*.* [10] evaluated maxillary dental arch changes in classes I and II malocclusion patients treated with a TPA during the mixed dentition followed by full fixed appliances in the permanent dentition. Most of the dental arch changes achieved at the end of the treatment, remained stable after an average 6.7-year follow-up. It was noted though that in some patients, relapse occurred ranging from 0.5 to 2 mm. However, occlusal and cephalometric differences between patients showing stability and those having relapse were not investigated. It would be therefore clinically relevant to better understand what initial occlusal characteristics may be good predictors of long-term stability under this treatment approach.

Therefore, the aim of this retrospective study was to identify which dental and/or cephalometric variables were predictors of long-term maxillary dental arch stability in patients that underwent treatment with a TPA during the mixed dentition followed by full fixed appliances in the permanent dentition.

## Methods

**Fig. 1:**
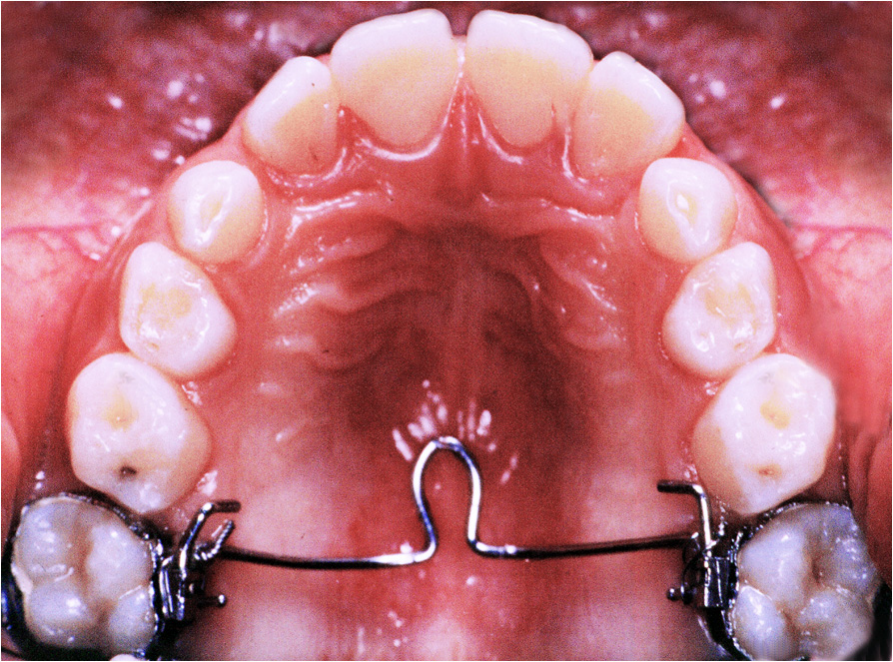
TPA applied to the first molars

Appropriate ethical approval was granted by the Health Research Ethics Board of University of Alberta (Pro00044194), by Burlington Growth Center (BGC) at the University of Toronto and by the Health Research Ethics Board of the Second University of Naples (No. 12554). Dental casts and lateral cephalograms of 36 consecutively treated patients (14 boys and 22 girls), gathered from a private orthodontic practice in Naples, Italy (L.P.), were considered. This same sample has been previously reported while answering a different clinical question [10]. Available records included data from before TPA treatment (*T*_0_), after TPA treatment (Fig. 1) but before full fixed appliances (*T*_1_), after full fixed appliance treatment (*T*_2_), and a minimum of 3 years after full fixed appliances’ removal (*T*_3_). Included patients were divided into stable and relapse groups based on the long-term presence or not of relapse (no crowding or more than 0.1 mm of total crowding) (Table 1). The control group was obtained from the BGC sample and matched as closely as possible (age, sex, malocclusion) with the treated individuals.

**Table 1:** Characteristics of samples

Group	Number			Average age (year/month)				Crowding (mm)			
	Total	Male	Female	*T*_0_	*T*_1_	*T*_2_	*T*_3_	*T*_0_	*T*_1_	*T*_2_	*T*_3_
TREATED	36	14	22	9.25	11.23	13.41	20.17	−4.65	−0.35	0.00	−0.47
Stable	18	6	12	9.34	11.39	13.62	19.51	−3.64	0.19	0.00	0.00
Relapse	18	7	11	9.15	11.06	13.20	20.82	−5.67	−0.89	0.00	−0.94

Included subjects had:

- Class I or II malocclusions,
- Mild to moderate crowding with need for lip support,
- Mixed dentition,
- Under 9 years of age at *T*_0_, and
- A cervical vertebral maturation (CVM) of 1 or 2 at *T*_0_.

None of the included patients had previous orthodontic treatment, skeletal posterior crossbite, and craniofacial anomalies or required an extraction treatment.

### Treatment protocol

Treatment included three phases. During the first phase, in the mixed dentition, a TPA was used to eliminate crowding by slight molar expansion and rotation. This TPA was initially cemented passively. During the second visit, the TPA was activated to achieve molar derotation with a 1-mm transverse expansion. During the second phase, in the permanent dentition, standard edgewise fixed appliances according to the Tweed-Merrifield technique (0.022 × 0.028 in.) were used to correct the residual crowding and to detail the occlusion as needed. The TPA was left passively during this phase to reinforce anchorage. During the retention phase, for about 2 years, an upper Hawley retainer was used. Therefore, the long-term follow-up without any retention appliance was of at least 1 year.

### Measurements

**Fig. 2:**
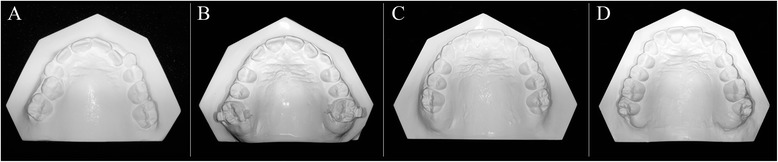
Upper dental casts at the four time periods. **a** Before treatment. **b** Post-TPA. **c** Post-fixed appliances. **d** Follow-up. * Figures are from Raucci et al [10] courtesy of The Angle Orthodontist

For the dental cast analysis, a black 2H pencil with a 0.5-mm tip was used to mark the maxillary anatomic landmarks [11] at the four time periods (Fig. 2). A digital caliber was then used to measure intercuspid width, interpremolar width, intermolar width, arch length, perimeter, and crowding.

**Fig. 3:**
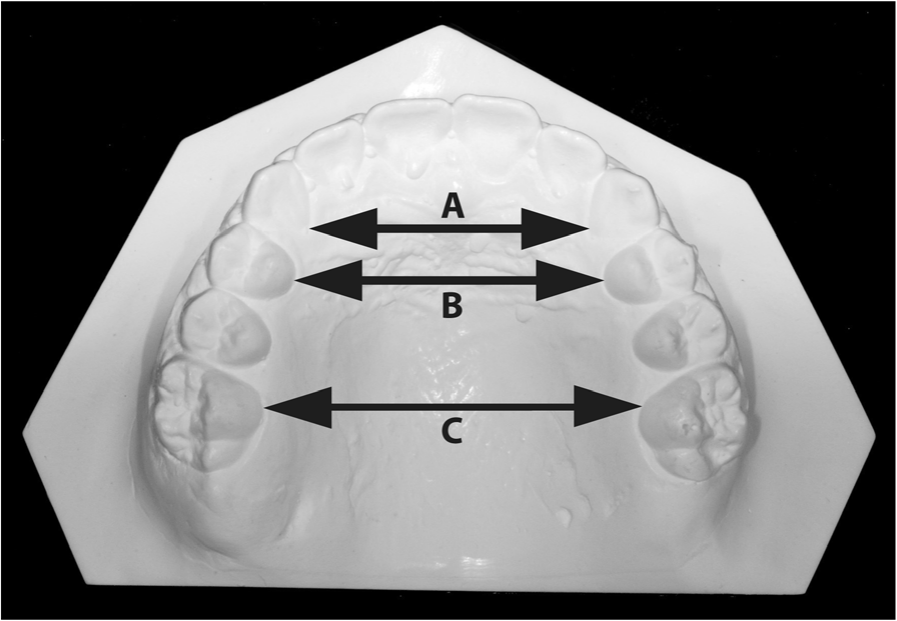
Arch width measurements. **a** Intercanine width. **b** Interpremolar width. **c** Intermolar width. *Figures are from Raucci et al [10] courtesy of The Angle Orthodontist

Intercuspid, interpremolar, and intermolar widths were measured between inner lingual points on the gingival margin of the deciduous or permanent canines, first deciduous molars or premolars, and first molars (Fig. 3).

**Fig. 4:**
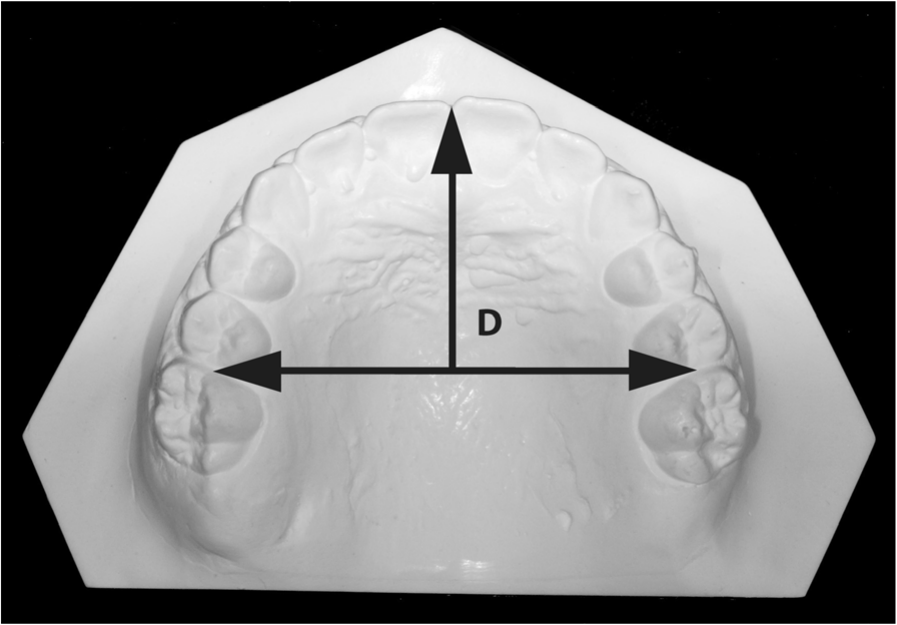
Arch length measurement (*D*), as the perpendicular distance from the most facial point on the most prominent central incisor to a line constructed between contact point mesial to the permanent first molars. *Figures are from Raucci et al [10] courtesy of The Angle Orthodontist

Arch length was measured as the perpendicular distance from the most facial point on the most prominent central incisor to a line constructed between contact point mesial to the permanent first molars (Fig. 4).

**Fig. 5:**
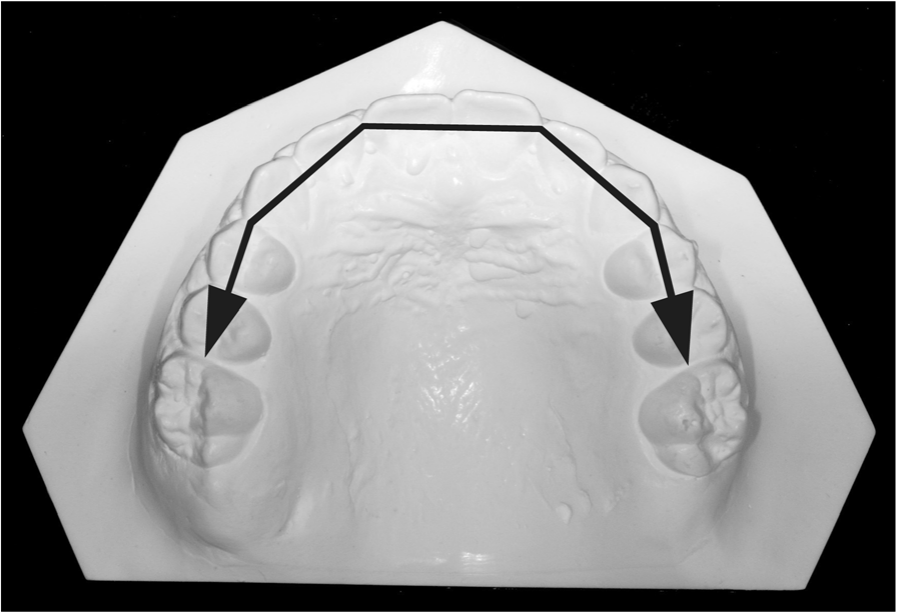
Arch perimeter measurement, as the sum of the segment lengths connecting contact point mesial to the permanent first molars. *Figures are from Raucci et al [10] courtesy of The Angle Orthodontist

Perimeter was evaluated as the sum of the distances between points on the mesial aspect of the permanent first molars, on the distal side of the canines and central incisors (Fig. 5). Unerupted teeth were represented by a point halfway between the adjacent permanent teeth centered buccolingually on the alveolar process.

Crowding was evaluated as tooth size-arch length discrepancy. Any crowding (>0.1 mm) at the end of follow-up observation (*T*_3_) was considered relapse.

The four casts of each patient were marked consecutively to ensure that the locations of all the landmarks were as identical as possible at each time period.

For the cephalometric analysis, only maxillary incisor inclination to palatal plane was considered.

### Statistical analysis

The statistical package for the Social Sciences (version 22; SPSS, Chicago, III) was used for data analysis.

For data description, mean and standard deviation (SD) were used for continuous variables, while frequencies were used for categorical variables.

A binary logistic regression analysis was performed to determine the impact of the dental cast and cephalometric measurements, as predictive variables, at *T*_1_ and their changes between *T*_0_ and *T*_1_ on the occurrence of relapse at *T*_3_ with a binary response (relapse vs*.* stable).

Different factors were further analyzed as determinants of relapse by multivariate logistic regression analysis.

A *p* value of less than 0.05 was considered statistically significant.

## Results

Reliability of measured values for both treatment and control groups was tested using measurement error (ME) calculated via Dahlberg’s formula (Table 2). All differences were relatively small, not likely clinically significant.

**Table 2:** Error of method values

Variables (all mm except U1/PP in degrees)	*T*	*C*
Intercuspid width	0.14	0.19
Interpremolar width	0.12	0.12
Intermolar width	0.17	0.15
Perimeter	0.18	0.08
Length	0.13	0.17
Crowding	0.16	0.03
U1/PP	0.04	0.06

Differences for the measurements between groups at *T*_0_, *T*_1_, *T*_2_, and *T*_3_ can be found in the previous publication [10].

A logistic regression was performed to ascertain the effects of maxillary arch widths, perimeter, and length, as well as crowding and upper incisor inclination at *T*_1_ on the likelihood that participants have long-term stable orthodontic treatment. The model explained 42.7 % (Nagelkerke R2) of the variance in treatment stability and correctly classified 72.2 % of the sample. Of the seven predictor variables, only upper anterior crowding (*p* = 0.029) was statistically significant (Table 3). For every millimeter of decreased crowding at *T*_1_ (after TPA treatment/before starting the fixed orthodontic treatment), there was an increase of 3.57 times in the odds of having stability.

**Table 3:** Logistic regression model predicting likelihood of the stability of the orthodontic treatment

	*B*	S.E.	Wald	*df*	*p* values	Odds ratio	95 % CI for odds ratio	
							Lower	Upper
Intercuspid width	−0.114	0.409	0.077	1	0.781	0.892	0.400	1.990
Interpremolar width	0.215	0.286	0.566	1	0.452	1.240	0.708	2.173
Intermolar width	0.035	0.308	0.013	1	0.911	1.035	0.566	1.892
Arch perimeter	−0.197	0.156	1.586	1	0.208	0.821	0.605	1.116
Arch length	−0.096	0.211	0.206	1	0.650	0.909	0.600	1.375
Crowding	1.277	0.586	4.747	1	0.029	3.585	1.137	11.306
U1/PP	0.120	0.094	1.650	1	0.199	1.128	0.939	1.355
Gender	0.931	1.007	0.856	1	0.355	2.538	0.353	18.258
Constant	−0.160	14.947	0.000	1	0.991	0.852		

Binary logistic regression analysis was also carried out to determine the impact of changes in variables between *T*_0_ and *T*_1_ on the treatment stability at *T*_3_; however, no significant association was detected. Two reasons could be attributed to the limited significant association of variables in the models: a small sample size and the high correlation between the variables. Therefore, a principal component analysis was performed to reduce the number of correlated variables, yet the limited sample size adequacy compromised the reliability of this test.

## Discussion

Our results increase our understanding of the long-term effects of a non-extraction orthodontic treatment using a TPA followed by fixed appliances. The initial treatment response was the elimination of the crowding identified at *T*_0_ [10], but as expected, perfect stability is utopic. By separating cases that demonstrated stability vs*.* unstability, the study goal was to determine which measured variables could predict stability in a clinical meaningful way. Only crowding in the upper arch at *T*_0_ was predictive.

In this sample, a high percentage of intercanine (93 %), interpremolar (96 %), and intermolar (96 %) widths and perimeter (89 %) increases were maintained after the retention period. A slight tendency toward relapse was detected with a small amount (0.47 mm) of crowding, but regardless of 4.18 mm of the initial crowding remained resolved [10]. In this sample, dentoalveolar compensation by proclination of upper incisors was avoided. This can be extrapolated by the lack of significant arch length increase.

After an average 6.7-year follow-up, 18 patients (50 %) showed relapse. According to the prediction, model maxillary crowding at *T*_0_ and *T*_1_ (5.67 and 0.89 mm in relapsed and 3.64 and −0.19 mm in stable groups, respectively) seemed to be the best predictor of relapse. The more crowding before treatment, the more relapse will occur (3.6 odds increase per millimeter).

It was found that the larger the dental expansion, the larger the relapse toward the starting position. We hypothesize that if the attained correction could be produced by physiological and not mechanical expansion, the relapse may be limited. It has been previously suggested that it is important to work with and not against the soft tissue equilibrium (cheek, lip, and tongue pressures) [12, 13].

The observed stability may also be the result, at least partially, of a good final intercuspation. But it has to be noted that in some cases, relapse occurred even with good intercuspidation.

When considering the available literature, a direct comparison of the results with other studies is difficult because of different appliances, sex, ages and ethnic group of the subjects, length of treatment, and method of analysis [3, 14, 15].

Occlusal changes in patients treated with TPA in mixed dentition, followed by fixed appliances, have been rarely documented. Except for a few case reports, TPA investigations have been mainly performed in vitro [16–22]. Only two [7, 23] have been carried out on patients. A smaller increase of the maxillary intermolar width of around 1 mm was reported in mixed dentition patients treated with TPA activated without expansion [7]. In the other study [23–25], patients were treated for posterior crossbite correction for 1 year without the control group and follow-up. Neither study is directly comparable. Currently, no long-term studies have been reported. The present study is the first of his kind.

### Limitations

The average 6.7-year follow-up included the retention period (approximately 2 years). From the total sample, seven patients (19 %) were only 1 year out of retention.

The findings need also to be interpreted cautiously for the lack of comparison to concurrent untreated controls. How much historical controls are equivalent may be controversial [26].

The definition of “crowding” is ambiguous. Crowding in this study was considered the tooth size-arch length discrepancy. The current definition of relapse was 0 mm. This may be too conservative, as some may not consider even 1–2 mm as clinically meaningful crowding.

The utilized TPA was made of round stainless steel wire. The use of a TMA alloy further reduces the applied force by 60 % [16]. The impact of this is unknown.

Finally, the results of this study are based only on a sample of patients treated without extractions.

## Conclusions

The best predictor of relapse was maxillary crowding before orthodontic treatment. The odds of relapse long-term (>3 years after full orthodontic treatment) increase by 3.6 times for every millimeter of crowding corrected during TPA treatment.
